# Surgical Treatment for Spinal Tuberculosis without Elevation of Inflammatory Biomarkers at the Initial Visit Mimicking Spinal Metastasis

**DOI:** 10.1155/2020/8873170

**Published:** 2020-08-25

**Authors:** Takuya Sakamoto, Hiroshi Takahashi, Junya Saito, Yasuo Matsuzawa, Yasuchika Aoki, Arata Nakajima, Masato Sonobe, Yorikazu Akatsu, Manabu Yamada, Yuki Akiyama, Tatsunori Iwai, Keita Yanagisawa, Yasuhiro Shiga, Kazuhide Inage, Sumihisa Orita, Yawara Eguchi, Satoshi Maki, Takeo Furuya, Tsutomu Akazawa, Masao Koda, Masashi Yamazaki, Seiji Ohtori, Koichi Nakagawa

**Affiliations:** ^1^Department of Orthopaedic Surgery, Toho University Sakura Medical Center, 564-1, Shimoshizu, Sakura City, Chiba 285-8741, Japan; ^2^Department of Orthopaedic Surgery, Faculty of Medicine, University of Tsukuba, 1-1-1, Tennodai, Tsukuba City, Ibaraki 305-8575, Japan; ^3^Department of Internal Medicine, Toho University Sakura Medical Center, 564-1, Shimoshizu, Sakura City, Chiba 285-8741, Japan; ^4^Department of Orthopaedic Surgery, Eastern Chiba Medical Center, 3-6-2, Okayamadai, Togane City, Chiba 283-8686, Japan; ^5^Department of Orthopaedic Surgery, Chiba University Graduate School of Medicine, 1-8-1, Inohana, Chuoku, Chiba City, Chiba 260-8677, Japan; ^6^Department of Orthopaedic Surgery, St. Marianna University School of Medicine, 2-16-1, Sugao, Miyamae, Kawasaki City, Kanagawa 216-8511, Japan

## Abstract

Here, we report a case of spinal tuberculosis without elevation of C-reactive protein (CRP) at the initial visit mimicking spinal metastasis. A 70-year-old woman developed progressive paraplegia without a history of injury and came to our hospital for evaluation. Severe compression to the spinal cord with osteolytic destruction of the spinal vertebrae at T6-7 was observed without elevation of CRP. A T4-9 posterior decompression and fusion were performed. Although the pathology revealed no malignant tumor cells, a positron emission tomography-computed tomography (PET-CT) showed upregulation of the thyroid gland and aspiration cytology revealed a thyroid carcinoma. Thus, we diagnosed her with spinal metastases from thyroid carcinoma. Conservative treatment was chosen with the hope of a significant neurologic recovery; however, 9 months after the primary surgery, she returned to our hospital with reprogressive paraplegia. In addition to progression of osteolytic changes to the T5-7 vertebrae, a coin lesion on the right side of the lung and elevation of CRP were observed. Finally, we diagnosed her with spinal tuberculosis based on the results of a CT-guided needle culture. Two-stage surgeries (posterior and anterior) were performed in addition to administering antituberculosis medications. At the 1-year postoperative follow-up evaluation, both neurologic function and laboratory data were improved with T5-9 complete fusion. It is difficult to determine based on imaging findings alone whether osteolytic vertebrae represent spinal metastases or tuberculosis. Even though inflammatory biomarkers, such as CRP, were not elevated, we should consider the possibility of not only spinal metastases but also tuberculosis when planning surgery involving osteolytic vertebrae. In addition, the combination of neurological, imaging, and pathological findings is important for the diagnosis of spinal tuberculosis.

## 1. Introduction

Spinal tuberculosis is a relatively rare condition which accounts for <1% of the total number of tuberculosis cases [1]. Usually, inflammatory biomarkers, such as C-reactive protein (CRP), are elevated in patients with tuberculosis [2]. Herein, we report a patient with spinal tuberculosis who required surgery, but did not have an elevated CRP at the initial visit, and was therefore thought to have spinal metastases.

## 2. Case Report

**Figure 1:**
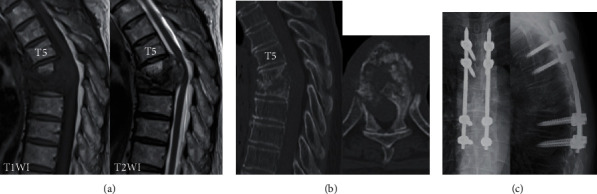
Imaging findings at the initial visit. (a) MRI sagittal imaging. T1 low and T2 iso-to-low extradural mass formation and severe compression to the spinal cord were observed. (b) CT sagittal reconstruction and axial imaging at T6. Osteolytic destruction of T6 and T7 vertebrae was observed. (c) X-ray just after the primary surgery.

**Figure 2:**
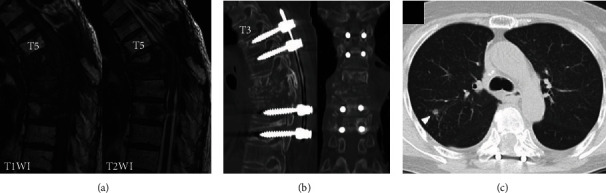
Imaging findings 9 months after surgery. (a) MRI sagittal imaging. (b) CT sagittal and coronal reconstruction. All the pedicle screws were loosened with the progression of the T6-7-8 vertebrae. (c) Chest CT. Arrowhead shows the coin lesion.

**Figure 3:**
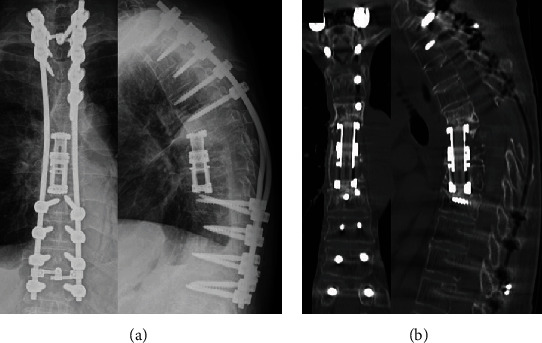
Imaging findings at the final follow-up. (a) X-ray. T1-12 posterior fusion and T6-7-8 corpectomy and anterior reconstruction with an expandable case were performed. (b) CT sagittal and coronal reconstruction. Complete bony fusion was observed inside the cage.

A 70-year-old Japanese woman who was previously healthy developed progressive paraplegia without a history of injury in the last 2 months. She thus came to our hospital for evaluation. The neurologic findings showed bilateral lower extremity muscle weakness (American Spinal Injury Impairment Scale C level) and severe hypalgesia and paresthesia at the T6-7 level. Severe compression to the spinal cord by osteolytic destruction of spinal vertebrae at T6-7 was demonstrated by computed tomography (CT) and magnetic resonance imaging (MRI) (Figures 1(a) and 1(b)). There were no fevers, and the laboratory data showed no elevation in the white blood cell count (WBC; 5450/*μ*l) or CRP (0.23 mg/dl). The patient accepted emergency surgery for the progressive paraplegia. A T4-9 posterior decompression and fusion with a T6-7 laminectomy were performed (Figure 1(c)). Although the pathology from the extirpated pedicle and lamina revealed inflammatory cell infiltrate without malignant tumor cells, the positron emission tomography-computed tomography (PET-CT) showed upregulation of the thyroid gland and aspiration cytology showed papillary adenocarcinoma of the thyroid. Thus, she was diagnosed with spinal metastases from a thyroid carcinoma. Although we explained the additional treatment of total spondylectomy (anterior and posterior) after the thyroidectomy, she declined additional surgery because the paraplegia improved significantly; however, 9 months after the primary surgery, she returned to our hospital with reprogressive paraplegia. Spinal cord compression by progression of osteolytic destruction of the T5-6-7 vertebrae with loosening of all pedicle screws was noted on MRI and CT (Figures 2(a) and 2(b)). The laboratory data showed elevation of CRP (11.64 mg/dl) without elevation of WBC (6470/*μ*l). Furthermore, a coin lesion in the right lung was observed (Figure 2(c)). Tuberculosis was suspected, and a CT-guided needle biopsy and culture were performed by the radiologist. Although a polymerase chain reaction was negative, *Mycobacterium tuberculosis* was detected from tissue culture. As a result, spinal tuberculosis was diagnosed. Two-stage surgeries (posterior and anterior) were performed, and antituberculosis medications (isoniazid, rifampicin, pyrazinamide, and ethambutol) were administered. In the first stage, a T1-10 posterior decompression and fusion with posterior drainage were performed. In the second stage, a T6-7-8 corpectomy, anterior drainage, and reconstruction with an expandable cage were performed (Figure 3(a)). Both neurologic function and laboratory data gradually improved after surgery. At the final follow-up 1 year after surgery, she could walk with a crutch, the laboratory data had returned to a normal level (CRP: 0.07 mg/dl), and complete fusion of T5-9 was observed on CT that showed healing of the spinal tuberculosis (Figure 3(b)).

## 3. Discussion

Generally, in the diagnosis of an osteolytic vertebra, it should be considered whether the diagnosis is spinal metastasis or spondylodiscitis. In contrast, spinal tuberculosis is a relatively rare condition compared with pyogenic spondylodiscitis. A past report of differential diagnosis between pyogenic spondylodiscitis and spinal tuberculosis indicated that the presence of fever, high WBC (>10000/*μ*l), and high CRP (>5 mg/dl) suggests pyogenic spondylodiscitis [3]. The noteworthy point is that our case revealed no elevation of CRP at the initial visit. Generally, biomarkers such as WBC and CRP were used to diagnose and evaluate the treatment for pulmonary tuberculosis [2]. In spinal tuberculosis, CRP elevation is mild compared with pyogenic spondylodiscitis [3]; however, no elevation of CRP is an extremely rare condition considering the severe osteolytic destruction of the spinal vertebrae. Thus, we initially did not suspect spinal tuberculosis but rather considered metastases. In fact, spinal tuberculosis is sometimes incorrectly diagnosed as spinal metastases, even with MRI or PET-CT [1, 4, 5]. However, in the diagnosis of spinal tuberculosis, the pathological finding is also important [6]. In this case, although the pathology during the first surgery did not reveal caseous necrosis, the inflammatory cell infiltrate was observed. The recent report indicated that the multiple characteristics like granulomatous inflammation in addition to caseous necrosis were observed in patients with spinal tuberculosis [7]. We should have considered the possibility of spinal tuberculosis with the result of pathology during the first surgery. Ultimately, the combination of neurological, imaging, and pathological findings is important for the diagnosis of spinal tuberculosis.

To treat spinal tuberculosis, an implant is acceptable because the biofilm formation is not severe in tuberculosis compared with other bacteria [8]. The prognosis is relatively good if adequate treatment was performed. An anterior-to-posterior or posterior-to-anterior procedure with instrumentation is recommended for patients with a small loss to correct, and the healing time is shorter [9]. In this case, we chose posterior and anterior surgery and used an expandable cage for anterior reconstruction, and complete healing was observed.

In conclusion, it is difficult to determine by imaging findings alone whether osteolytic vertebrae represent spinal metastases or tuberculosis. Even though inflammatory biomarkers such as CRP are not elevated, we should consider the possibility of not only spinal metastases but also tuberculosis when planning treatment or surgery of such osteolytic vertebrae. In addition, the combination of neurological, imaging, and pathological findings is important for the diagnosis of spinal tuberculosis.
